# Co-existing obstructive sleep apnea reduces Nuss surgery efficacy in pectus excavatum

**DOI:** 10.1371/journal.pone.0277494

**Published:** 2022-11-11

**Authors:** Yi-Chih Huang, Yeung-Leung Cheng, Wen-Lin Su, Chou-Chin Lan, Yao-Kuang Wu, Mei-Chen Yang

**Affiliations:** 1 Division of Pulmonary Medicine, Department of Internal Medicine, Taipei Tzu Chi Hospital, Buddhist Tzu Chi Medical Foundation, New Taipei, Taiwan; 2 School of Medicine, Tzu Chi University, Hualien, Taiwan; 3 Division of Chest Surgery, Department of Surgery, Taipei Tzu Chi Hospital, Buddhist Tzu Chi Medical Foundation, New Taipei, Taiwan; SWEDEN

## Abstract

Nuss surgery is effective in correcting pectus excavatum (PE), with a recurrence rate of 1.2–27%. Re-do surgery is successful but still has a 6% failure rate. Patients with obstructive sleep apnea (OSA) experience repetitive PE-associated sternal depression during sleep. As the prevalence of OSA among PE patients is higher than the average, co-existing OSA in PE patients might negatively affect the efficacy of Nuss surgery. This study aimed to evaluate the impact of co-existing OSA on Nuss surgery in patients with PE. In total, 20 adult patients with PE only and 9 patients with PE and OSA were analyzed. Polysomnography was performed before Nuss surgery to evaluate OSA. Sternovertebral distance (SVD) and radiographic Haller index (RHI) were recorded before surgery and at 3, 6, and 24 months postoperatively. The results showed that percentage changes in SVD in patients with PE only at 3, 6, and 24 months postoperatively were significantly increased compared with those in the patients with PE and OSA (31.1% vs. 14.1% at 3 months; 37.5% vs. 21.4% at 6 months; 42.5% vs. 19.2% at 24 months). Meanwhile, percentage changes in RHI were significantly lower in patients with PE alone than in the patients with PE and OSA (-22.9% vs. -9.3% at 3 months; -27.9% vs. -18.7% at 6 months; -30.6% vs. -16.7% at 24 months). This study showed that co-existing OSA might reduce the efficacy of Nuss surgery for patients with PE. We recommend that patients with PE should be evaluated and treated for OSA before surgery to prevent surgical failure after bar removal.

## Introduction

Nuss surgery is safe and effective in correcting pectus excavatum (PE) [1]. The recurrence rate of PE after Nuss surgery ranges from 1.2% to 27% [2–6]. Secondary surgical repair (re-do surgery) is often indicated for primary surgical failure or recurrence, with an excellent success rate in patients with PE [7–12]. However, there is still a 6% failure rate in repeat surgery after bar removal [9]. Previous investigations have reported that predictors of surgical failure or recurrence are age [2.3], premature bar removal [2], presence of postoperative complications [4], immediate postoperative failure [3], asymmetrical pectus deformity [5], disease severity with a Haller index >4.0 [3, 5], and level of surgeons’ experience [2]. Obstructive sleep apnea (OSA) is more prevalent in patients with PE than in the general population [13], and patients with OSA might present with PE. In 2016, Ma et al. reported a case of a 5-year-old child with severe OSA and PE, in whom PE was resolved after surgical treatment to eliminate OSA [14]. This indicates that OSA is a risk factor for the development of PE; therefore, it is a predictor of surgical failure or PE recurrence. Our previous study [15] found that co-existing OSA in patients with PE could not be improved after Nuss surgery, suggesting that OSA might be a contributing risk factor for surgical failure or recurrence of PE. Therefore, to address the knowledge gap, this study aimed to evaluate the impact of co-existing OSA on the efficacy of Nuss surgery in patients with PE.

## Materials and methods

### Participants

**Fig 1:**
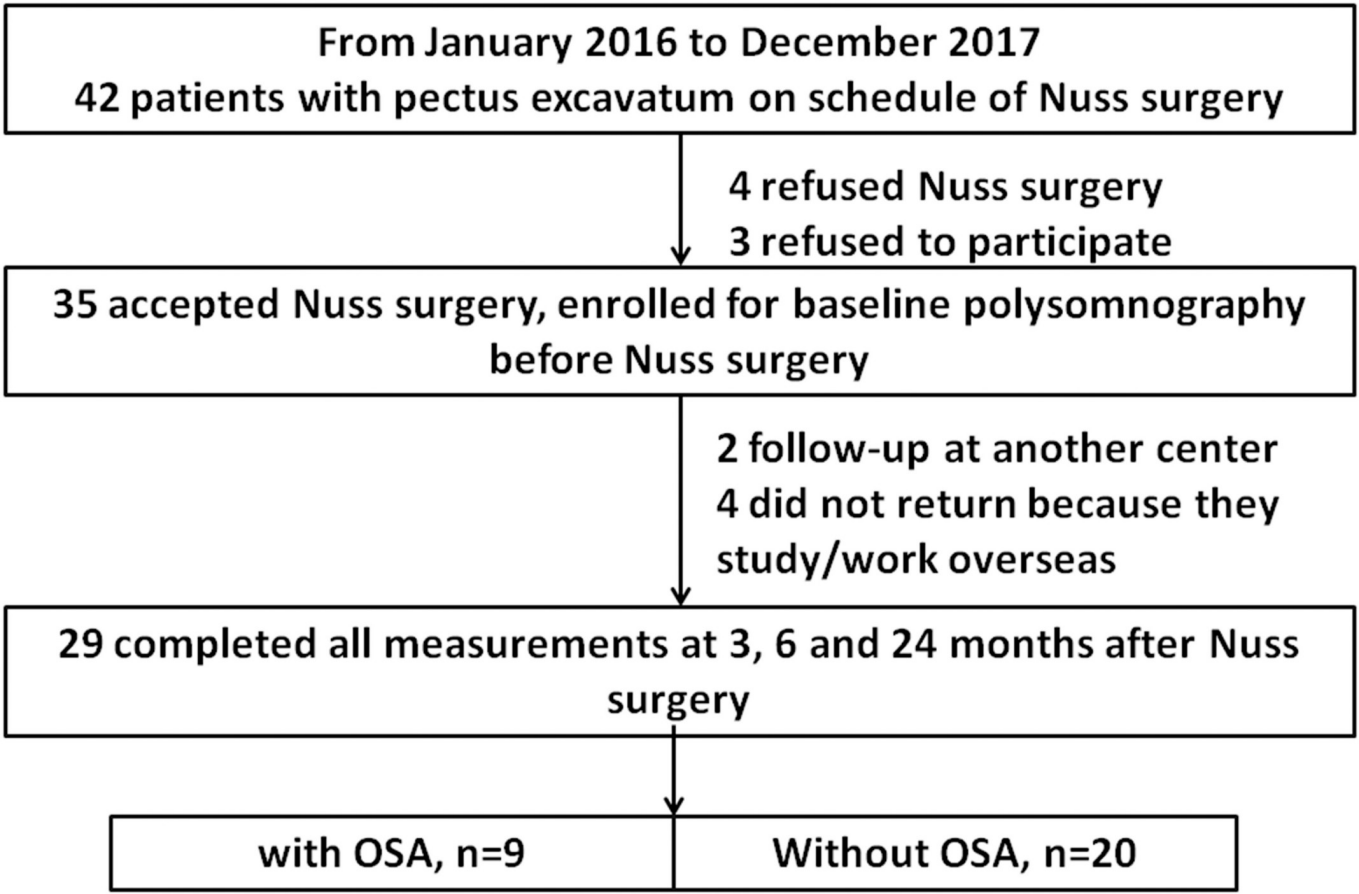
Flow chart of the study. Flowchart of the study participant selection process.

This was a prospective observational comparative study. A total of 42 adult patients with PE who were scheduled for Nuss surgery were recruited from January 2016 to December 2017 at Taipei Tzu Chi Hospital, New Taipei, Taiwan (Fig 1). The inclusion criteria were as follows: age 20–45 years; Haller index ≥3.0; no psychiatric or medical illness; and no use of psychoactive, hypnotic, or illegal drugs. The exclusion criteria were as follows: age < 20 years or > 45 years; Haller index <3.0; psychiatric or medical illness; and use of psychoactive, hypnotic, or illegal drugs. A total of four patients refused to undergo Nuss surgery, and three declined to participate in the study. Therefore, 35 patients were enrolled, and polysomnography (PSG) was performed on these patients before Nuss surgery to evaluate OSA. After Nuss surgery, two patients were followed-up at another medical center, and four did not return because of overseas work or study commitments. The remaining 29 patients completed all follow-up examinations at 3, 6, and 24 months after surgery and before bar removal and were included in the final analysis. Among the 29 patients, 20 had no OSA (apnea/hypopnea index [AHI] <5.0/h on PSG) and were assigned to the control group. The other nine had OSA (AHI ≥5.0/h on PSG) and were assigned to the study group. Participation was voluntary and written informed consent was obtained from all participants. The study was approved by the Institutional Review Board of Taipei Tzu Chi Hospital (protocol number: 04-XD15-056 and 09-XD-138).

### Measurements of the severity of pectus excavatum

We used the radiographic Haller index (RHI) and sternovertebral distance (SVD) to evaluate the efficacy of Nuss surgery since these parameters have been increasingly used and because of their advantage of less radiation exposure [3, 16]. Chest radiographs, including the anteroposterior and lateral views, were used to measure SVD and RHI. SVD was measured from the point of the most posterior projection of the sternum on the lateral view to the same vertebral level. The RHI was measured as the ratio of the transverse diameter to the anteroposterior diameter at the point of deepest chest wall depression [3, 16].

### Anthropometric characteristics

Data on patient characteristics, such as age, sex, smoking status, body height (BH), body weight (BW), body mass index (BMI), and Haller index [17], were collected at baseline.

### Polysomnography examination

Trained sleep technicians performed standard overnight PSG (Compumedics Profusion PSG 3, Abbotsford VIC, Australia). PSG recordings included movement monitoring using a position sensor, oxygen saturation monitoring using a pulse oximeter, evaluation of snoring using a microphone, and airflow measurement through the nose and mouth (using a thermistor and based on nasal pressure). Body position was identified using a trunk position sensor. Respiratory events were evaluated based on plethysmographic findings of the chest and abdomen. Each PSG recording lasted for at least 6 hours. Every 30-second epoch was manually scored by sleep technicians and rechecked by a sleep specialist. Sleep stage and respiratory events were scored according to the 2012 American Academy of Sleep Medicine criteria [18]. OSA was diagnosed when the AHI was ≥5/h.

### Statistical analysis

Data of categorical variables are presented as counts (%) and were analyzed using Pearson’s chi-square test with Fisher’s exact test. Continuous variables are presented as medians (quartile ranges) (Q1, Q3). The Mann–Whitney U test was used for between-group comparisons at baseline and at 3, 6, and 24 months after surgery. Spearman’s correlation was used to evaluate the correlation between the presence of OSA and the changes in SVD and RHI after surgery at 3, 6, and 24 months. Correlation was considered high, moderate, or low with a Spearman’s rho of >0.7, 0.4–0.7, or 0.2–0.4, respectively. All statistical assessments were two-sided (p<0.05). Statistical analyses were performed using IBM SPSS statistical software (version 24 for Windows, IBM Corp., Armonk, NY, USA).

## Results

**Fig 2:**
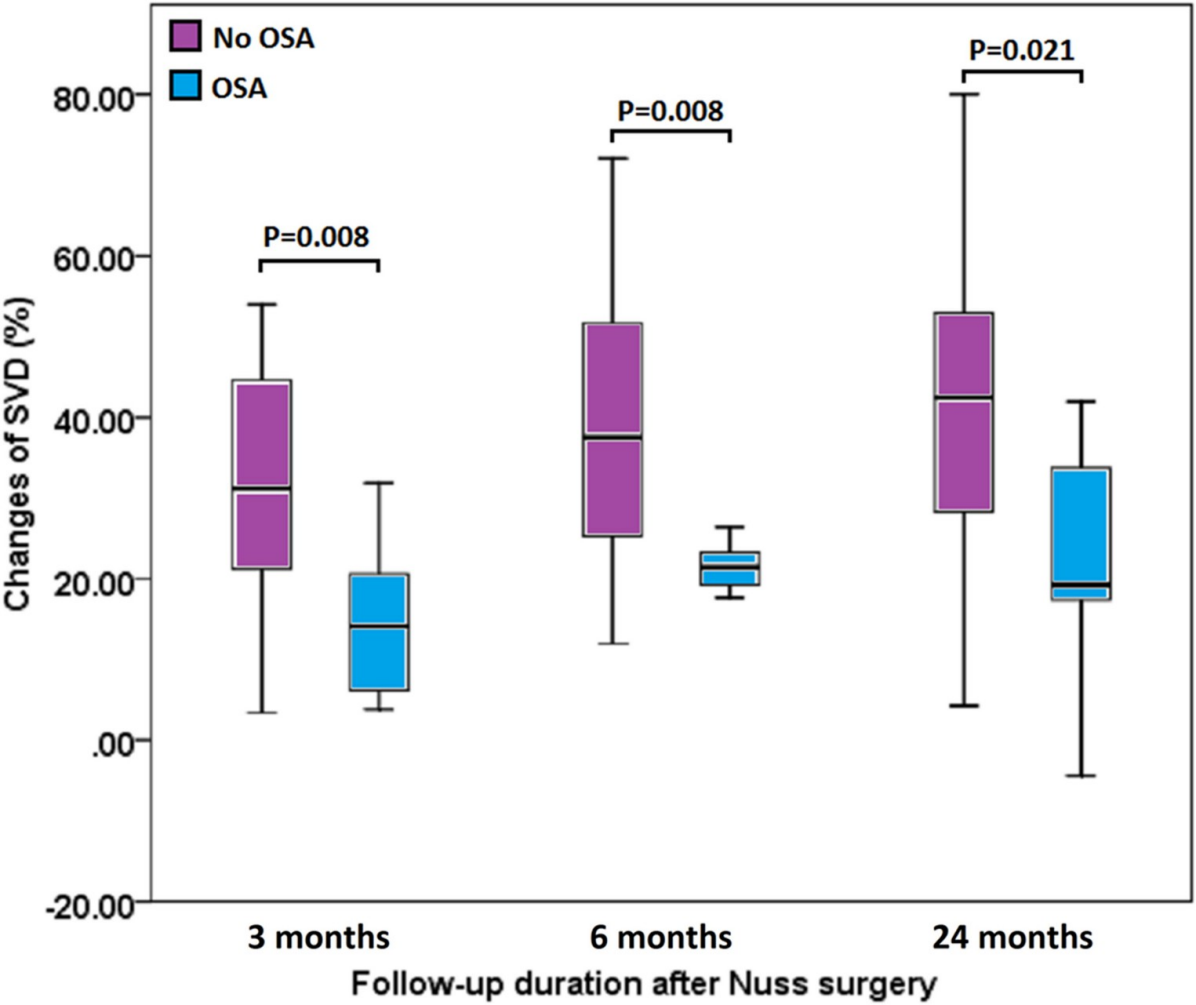
Comparisons of the percentage changes in sternovertebral distance. Comparisons of the percentage changes in sternovertebral distance (SVD) between pectus excavatum patients with obstructive sleep apnea (OSA) and those without OSA after surgery at 3, 6, and 24 months.

**Fig 3:**
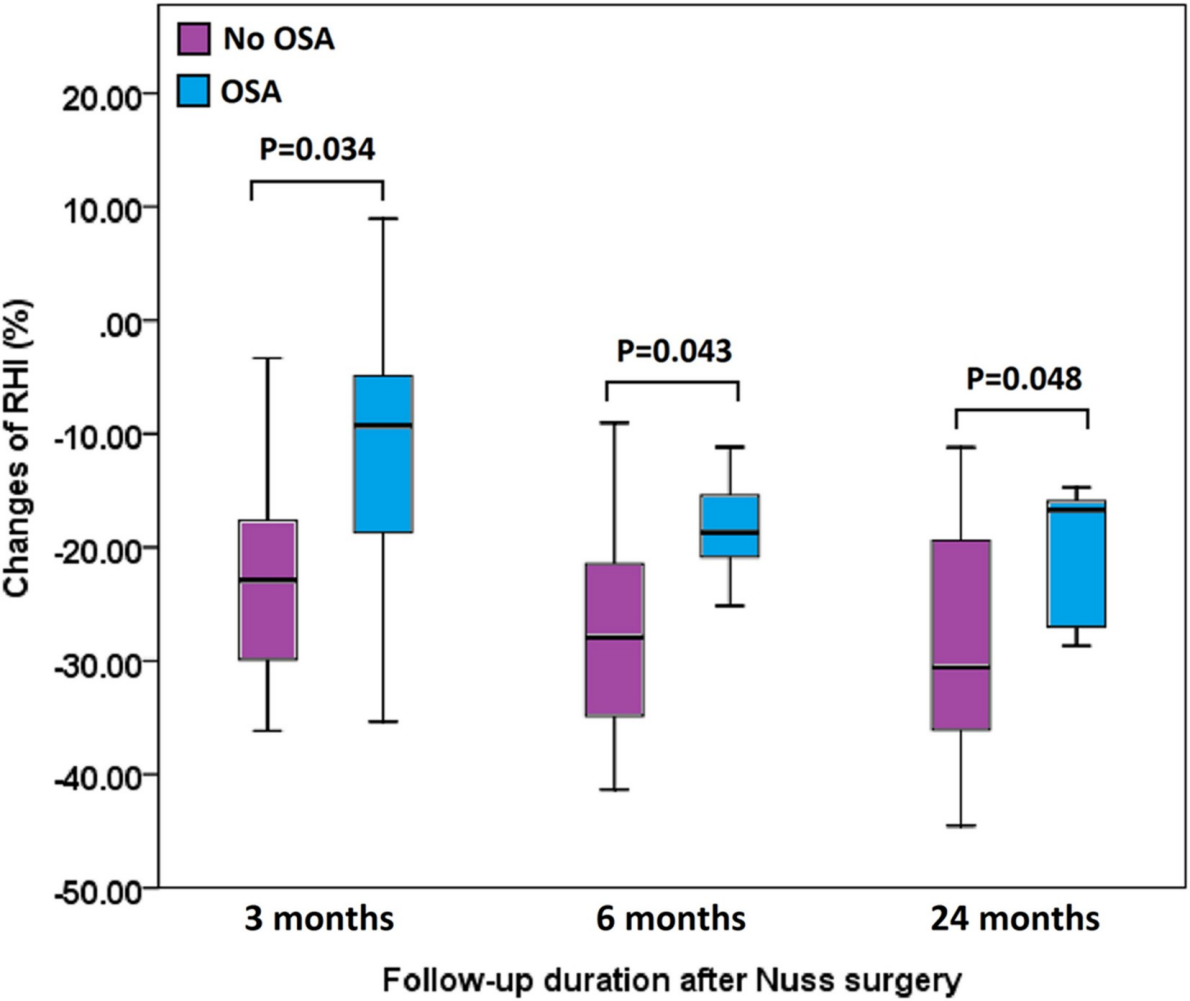
Comparisons of the percentage changes in radiographic Haller index. Comparisons of the percentage changes in radiographic Haller index (RHI) between pectus excavatum patients with obstructive sleep apnea (OSA) and those without OSA after surgery at 3, 6, and 24 months.

At baseline, patients in the study group were older than those in the control group (median age 31.0 vs. 23.0, p = 0.013) (Table 1). The AHI of the study group was higher than that of the control group (median AHI 11.3/h vs. 1.3/h, p = 0.000). There were no significant differences in sex, smoking status, Haller index, RHI, SVD, BH, BW, and BMI between both groups. After Nuss surgery, the improvement in SVD was less in the study group than in the control group (1.1 cm vs. 2.4 cm, p = 0.006, at 3 months; 1.8 cm vs. 2.7 cm, p = 0.006, at 6 months; and 1.5 cm vs. 3.1 cm, p = 0.021, at 24 months postoperatively), and the percentage of SVD improvement was less in the study group than in the control group (14.1% vs. 31.1%, p = 0.008, at 3 months; 21.4% vs. 37.5%, p = 0.008, at 6 months; and 19.2% vs. 42.5%, p = 0.021, at 24 months). The reduction in RHI in the study group was less than that in the control group 3 months after surgery (-0.3 vs. -0.9, p = 0.038), but there was no significant difference at 6 or 24 months. The percentage of RHI reduction was less in the study group than in the control group after surgery (-9.3% vs. -22.9%, p = 0.034, at 3 months; -18.7% vs. -27.9%, p = 0.043, at 6 months; and -16.7% vs. -30.6%, p = 0.048, at 24 months) (Table 1, Figs 2 and 3).

**Table 1:** Comparison of baseline characteristics and changes between patients with pectus excavatum only and patients with pectus excavatum and obstructive sleep apnea.

Characteristic	With OSA, n = 9	Without OSA, n = 20 N = 20	p-value
**Baseline characteristics**			
Sex			0.568^a^
Male, n (%)	7 (77.8%)	18 (90.0%)	
Female, n (%)	2 (22.2%)	2 (10.0%)	
Smoking status			1.000^a^ ^b^
Smoker, n (%)	0 (0%)	22 (10.0%)	
Non-smoker, n (%)	9 (100.0%)	18 (90.0%)	
Age, years	31.0 (25.0; 38.0)	23.0 (21.3; 24.8)	0.013*
Body height, cm	173.0 (160.5; 178.5)	173.5 (170.0; 179.1)	0.450
Body weight, kg	66.0 (52.0; 73.0)	61.0 (54.8; 64.8)	0.345
Body mass index, kg/m^2^	21.4 (19.2; 24.0)	20.0 (18.3; 21.3)	0.120
Haller index	3.5 (3.3; 3.9)	3.4 (3.2; 4.1)	0.850
RHI	3.3 (3.2; 3.5)	3.5 (3.1; 4.4)	0.637
SVD, cm	7.9 (7.4; 8.5)	7.7 (6.3; 8.5)	0.464
Apnea/hypopnea index, /h	11.3 (8.2; 43.2)	1.3 (0.5; 2.7)	0.000*
**Changes in SVD after surgery**			
△SVD_3m, cm	1.1 (0.5; 1.8)	2.4 (1.4; 2.7)	0.006*
△SVD_3m, %	14.1 (5.5; 21.8)	31.1 (20.6; 45.0)	0.008*
△SVD_6m, cm	1.8 (1.4; 2.2)	2.7 (1.9; 3.2)	0.006*
△SVD_6m, %	21.4 (18.4; 24.8)	37.5 (24.9; 52.8)	0.008*
△SVD_24m, cm	1.5 (1.3; 3.0)	3.1 (2.3; 3.7)	0.036*
△SVD_24m, %	19.2 (17.0; 34.7)	42.5 (27.3; 53.4)	0.021*
**Changes in RHI after surgery**			
△RHI_3m, cm	-0.3 (-0.7; -0.1)	-0.9 (-1.2; -0.5)	0.038*
△RHI_3m, %	-9.3 (-21.5; -4.3)	-22.9 (-30.2; -17.3)	0.034*
△RHI_6m, cm	-0.7 (-0.8; -0.4)	-1.0 (-1.4; -0.6)	0.077
△RHI_6m, %	-18.7 (-23.0; -13.3)	-27.9 (-35.1; -21.2)	0.043*
△RHI_24m, cm	-0.6 (-0.9; -0.5)	-1.1 (-1.5; -0.5)	0.109
△RHI_24m, %	-16.7 (-27.9; -15.3)	-30.6 (-36.0; -18.7)	0.048*

Comparison of baseline characteristics and the changes in sternovertebral distance and radiographic Haller index between pectus excavatum patients with obstructive sleep apnea and those without obstructive sleep apnea after surgery at 3, 6, and 24 months.

Abbreviations: OSA, obstructive sleep apnea; RHI, radiographic Haller index; SVD, sternovertebral distance; △ in value (cm), the value at baseline minus the value at 3, 6, and 24 months postoperatively; △ in percentage (%), percentage changes in the value from baseline to follow-up at 3, 6, and 24 months postoperatively.

^a^ Fisher’s exact test

^b^ the p-value might be incorrect because there were no smokers in the OSA group.

*Significant different with p<0.05 (two-wail)

**Fig 4:**
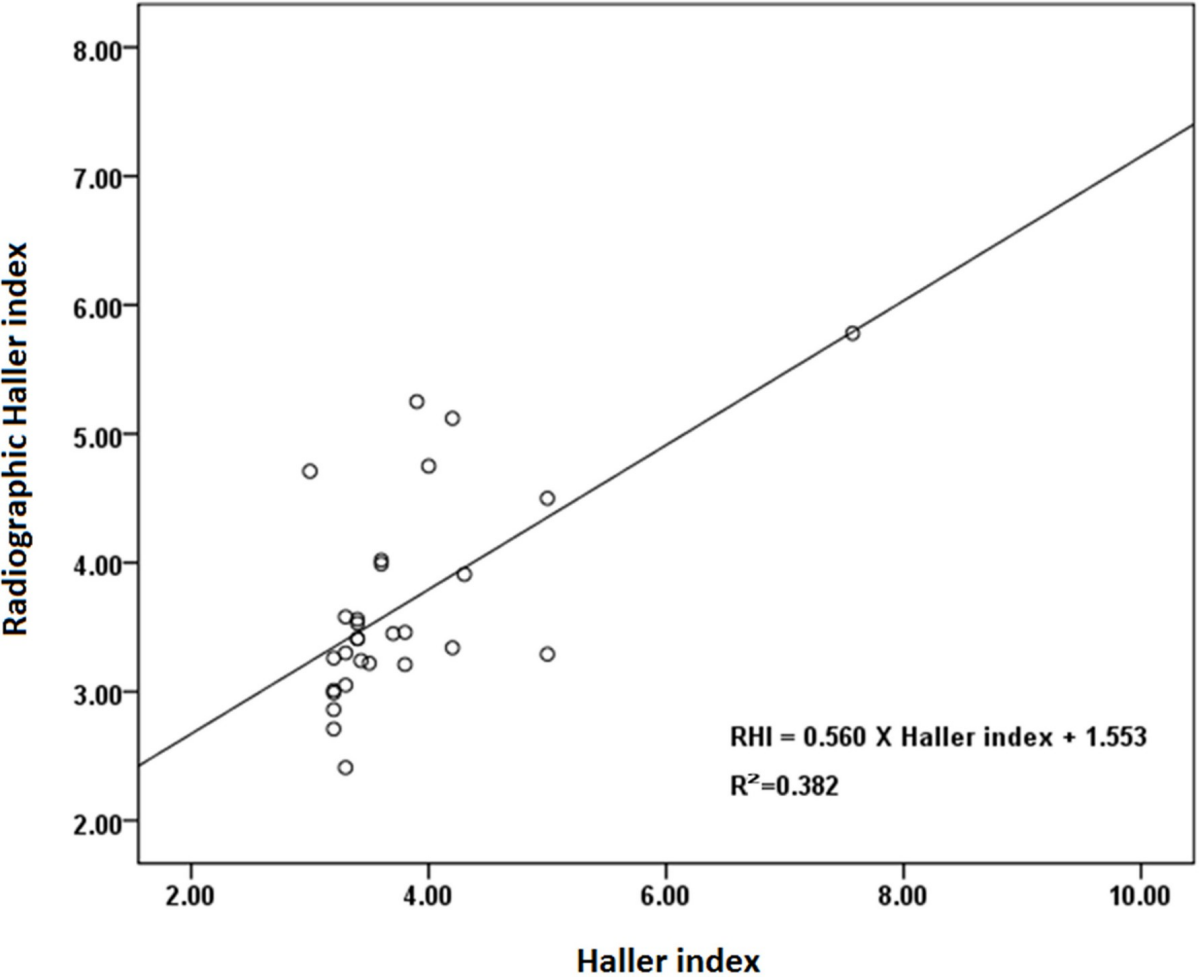
The relationship between Haller index and radiographic Haller index. The radiographic Haller index (RHI) was positively correlated with the Haller index.

**Fig 5:**
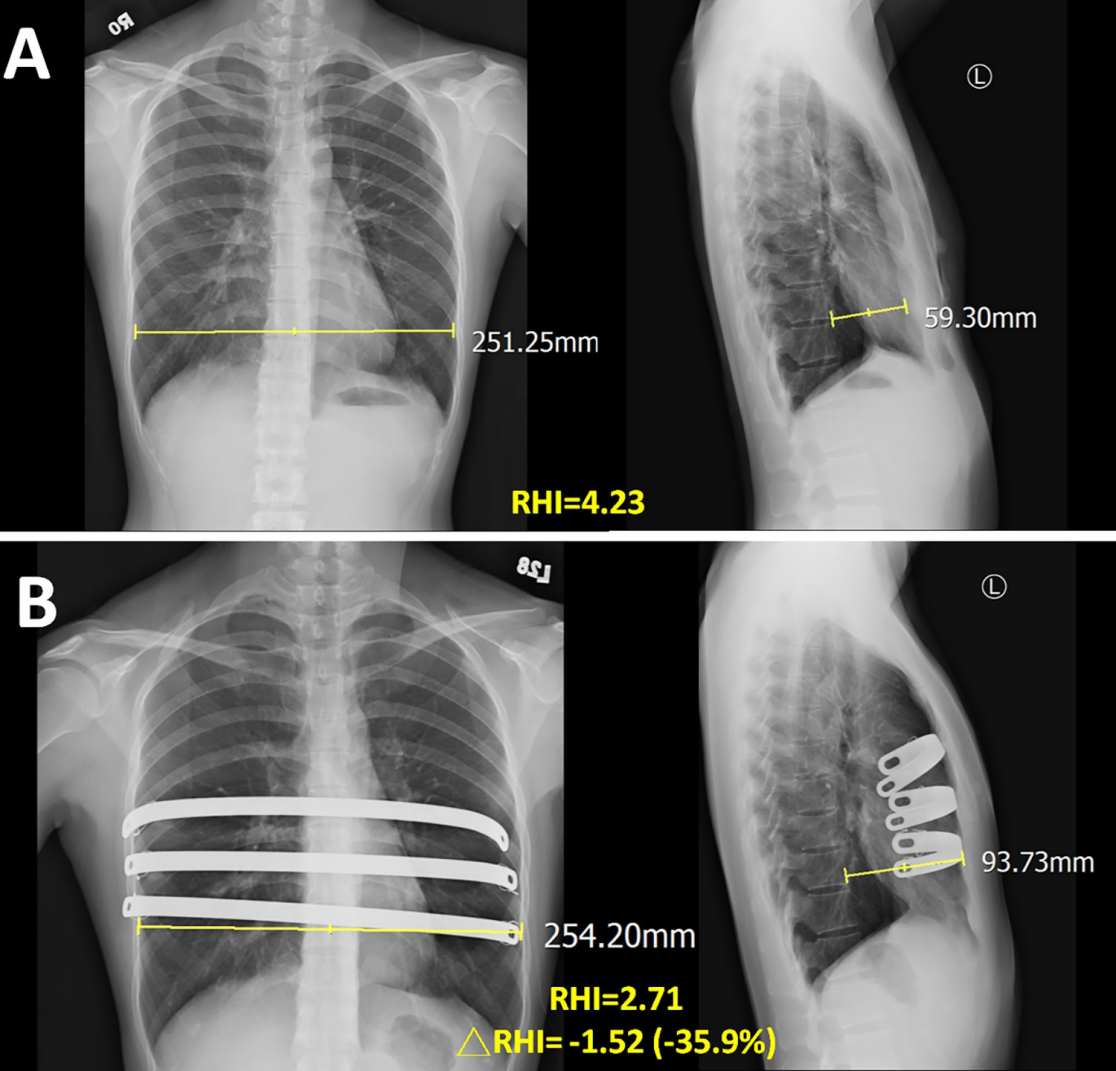
The changes of sternovertebral distance and radiographic Haller index in a patient with pectus excavatum only. (A) Before Nuss surgery, a patient with pectus excavatum only with a sternovertebral distance 59.3mm and a radiographic Haller index (RHI) 4.23. (B) After Nuss surgery 24 months, the sternovertebral distance increased to 93.7mm (increasing 58%) and the RHI decreased to 2.71 (decreasing 35.9%).

**Fig 6:**
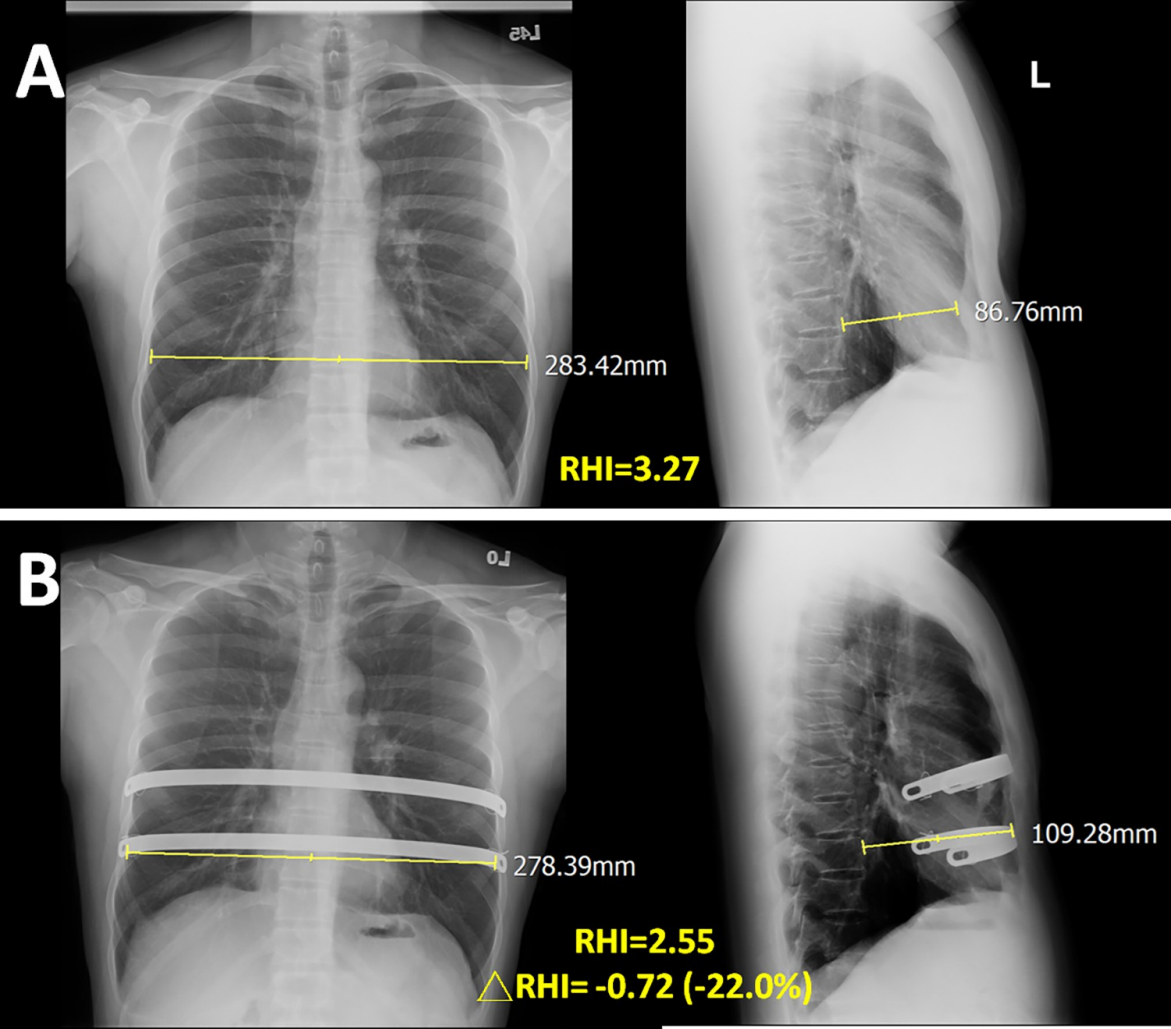
The changes of sternovertebral distance and radiographic Haller index in a patient with pectus excavatum and obstructive sleep apnea. (A) Before Nuss surgery, a patient with pectus excavatum and obstructive sleep apnea a sternovertebral distance of 86.8 mm, and a radiographic Haller index (RHI) of 3.27 mm. (B) After Nuss surgery at 24 months, the sternovertebral distance increased to 109.3 mm (25.9% increase) and the RHI decreased to 2.55 (22% decrease).

Spearman’s correlation analysis showed that co-existing OSA was negatively correlated with changes in SVD and RHI at 3, 6, and 24 months after surgery (Tables 2 and 3). The RHI of the study group was highly correlated with the Haller index (Fig 4). In a 24-year-old patient with PE only, the reduction in RHI at 24 months after surgery was 35.9% (Fig 5). However, in another 22-year-old patient with PE and OSA, the reduction in RHI at 24 months after surgery was only 22.0% (Fig 6).

**Table 2:** The correlations between co-existing obstructive sleep apnea and the changes in sternovertebral distance.

Spearman’s rho	OSA	△SVD_3m (%)	△SVD_6m (%)	△SVD_24m (%)	△SVD_3m (cm)	△SVD_6m (cm)	△SVD_24m (cm)
**OSA**	1.000						
**△SVD_3m, (%)**	-0.499*	1.000					
**△SVD_6m, (%)**	-0.499*	0.946*	1.000				
**△SVD_24m, (%)**	-0.437*	0.643*	0.734*	1.000			
**△SVD_3m, (cm)**	-0.517*	0.926*	0.863*	0.527*	1.000		
**△SVD_6m, (cm)**	-0.521*	0.865*	0.932*	0.637*	0.898*	1.000	
**△SVD_24m, (cm)**	-0.397*	0.471*	0.538*	0.899*	0.468*	0.543*	1.000

The Spearman’s correlations between co-existing obstructive sleep apnea and the changes in sternovertebral distance after Nuss surgery at 3, 6, and 24 months.

*Correlation is significant at the 0.05 level (2-tailed).

Abbreviations: OSA, obstructive sleep apnea; SVD, sternovertebral distance; △ in value (cm), the value at baseline minus the value at 3, 6, and 24 months postoperatively; △ in percentage (%), percentage changes in the value from baseline to follow-up at 3, 6, and 24 months postoperatively.

**Table 3:** The correlations between co-existing obstructive sleep apnea and the changes in radiographic Haller index.

Spearman’s rho	OSA	△RHI_3m (%)	△RHI_6m (%)	△RHI_24m (%)	△RHI_3m (value)	△RHI_6m (value)	△RHI_24m (value)
**OSA**	1.000						
**△RHI_3m, (%)**	0.401*	1.000					
**△RHI_6m. (%)**	0.383*	0.916*	1.000				
**△RHI_24m, (%)**	0.374*	0.796*	0.831*	1.000			
**△RHI_3m, (value)**	0.392*	0.966*	0.954*	0.800*	1.000		
**△RHI_6m, (value)**	0.334	0.903*	0.986*	0.836*	0.956*	1.000	
**△RHI_24m, (value)**	0.303	0.803*	0.849*	0.957*	0.851*	0.880*	1.000

The Spearman’s correlations between co-existing obstructive sleep apnea and the changes of radiographic Haller index after Nuss surgery at 3, 6, and 24 months.

*Correlation is significant at the 0.05 level (2-tailed).

Abbreviations: OSA, obstructive sleep apnea; RHI, radiographic Haller index; △ in value (cm), the value at baseline minus the value at 3, 6, and 24 months postoperatively; △ in percentage (%), the percentage changes in value from baseline to follow-up at 3, 6, and 24 months postoperatively.

## Discussion

This study demonstrated that co-existing OSA negatively affected the efficacy of Nuss surgery. To the best of our knowledge, this is the first study to evaluate the impact of OSA on Nuss surgery.

Laryngomalacia is associated with both OSA and PE [19–22]. Patients with OSA or laryngomalacia experience repeated upper airway obstruction resulting in chest retraction and sternum depression during inspiration, which mimics PE, and might eventually develop into PE if left untreated. In 1984, Lane reported a case of severe laryngomalacia presenting as PE, which resolved after surgical treatment for laryngomalacia [23]. In 2005, Avelino reported that 9.1% of patients with laryngomalacia had PE that needed supraglottoplasty [22]. In 2013, Schaerer also found an increased prevalence (6.6%) of PE among patients with laryngomalacia requiring supraglottoplasty [21].

In 1992, Castiglione et al. reported a very high prevalence of PE (82%) among 23 children with OSA [24]. In 2016, Ma et al. reported a case of a 5-year-old child with severe OSA who experienced aggravated severe sternum depression mimicking PE during an episode of upper airway infection [14]. His severe OSA and sternal depression improved after successful surgical treatment for OSA, but it did not correct the PE. In our previous study, we found that AHI in patients with PE did not improve with Nuss surgery [15]. If co-existing OSA cannot be treated adequately after Nuss surgery, then OSA-related sternal depression will repeatedly occur during sleep and might attenuate the efficacy of the surgery. Our results in this study also showed that patients with OSA had less improvement in SVD and RHI after Nuss surgery at 3, 6, and 24 months. Although OSA attenuated the efficacy of Nuss surgery in our study group, none of the patients were considered to have experienced primary surgical failure, and no repeat surgery was performed. This might be because we only followed up with patients before bar removal, and because the supporting effect of the surgical bars was still present. Follow-up data should be collected after bar removal to further evaluate the true impact of co-existing OSA on PE recurrence.

Continuous positive airway pressure (CPAP) is the first choice of treatment for OSA because it is non-invasive [25]. As OSA could be a risk factor for the development or deterioration of PE and for reducing the efficacy of Nuss surgery, it is worth screening for and treating with CPAP before considering Nuss surgery. It is also necessary to re-screen for OSA after bar removal or if PE recurs after surgical correction.

The Haller index, calculated from chest computed tomography (CT), represents the severity of PE. A pectus index >3.2 is a prerequisite for third-party insurance reimbursement for surgically corrective procedures [17]. Although the CT Haller index remains the primary method of preoperative imaging, it is rarely used postoperatively because of radiation exposure. Rattan et al. suggested that a limited “very low-dose” chest CT would suffice to assess the Haller index in most patients and implemented it as a protocol at their institution [26]. However, repeated radiation exposure is still problematic, especially considering that most patients with PE considering Nuss surgery are adolescents and young adults. The RHI was calculated using anteroposterior and lateral plain film radiographs and was strongly correlated with the CT Haller index with a very high Pearson correlation coefficient rho of 0.984 [27]. Due to the lower radiation dose, RHI has often been used as the primary tool for pre-and post-operative evaluation of PE, unless the patient is at high risk of complications [3, 16]. This study also showed a very high correlation between RHI and CT Haller index. Therefore, we believe that our results would be representative if we used the RHI to evaluate the efficacy of Nuss surgery.

This study had several limitations. First, the extremely small number of cases might have resulted in selection bias, and the results might not be representative of the entire population. Furthermore, we did not observe these patients after bar removal, and therefore, could not determine the outcome. Additionally, only adult patients aged 20–45 years were included, who were thought to have a higher recurrence rate of Nuss surgery than younger patients. Different results may be observed for patients aged < 20 years or > 45 years. Most of the patients who had undergone Nuss surgery in previously conducted studies were younger than our patients. Furthermore, we observed only the negative effects of OSA on Nuss surgery; however, we did not use CPAP to treat OSA in patients with PE after Nuss surgery to determine whether CPAP treatment for co-existing OSA could enhance the efficacy of Nuss surgery. Therefore, we could not conclude that OSA may contribute to surgical failure. Finally, none of our patients claimed surgical failure or needed re-do surgery by our surgeon at 24 months. Thus, the results can only support the conclusion that OSA has a negative effect on Nuss surgery, but the causal relationship between OAS and surgical failure or recurrence cannot be determined. All of these limitations indicate the need for larger, well-controlled prospective studies to document the causal relationship between OSA and PE and its impacts on Nuss surgery.

## Conclusions

The efficacy of Nuss surgery was significantly impaired by co-existing OSA. We suggest that it might be necessary to screen for OSA among patients with PE before Nuss surgery.
